# Case report: Novel phenotype in central 22q11.2 deletion syndrome

**DOI:** 10.1002/ccr3.2870

**Published:** 2020-10-18

**Authors:** Patrick Dideum, Luis Rohena, Janet Berg, Candace Percival

**Affiliations:** ^1^ Department of Pediatrics Brooke Army Medical Center Fort Sam Houston Texas

**Keywords:** 22q11.2 deletion, central 22q11 deletion, distal 22q11 deletion, failure to thrive, LCR22, microdeletion

## Abstract

Deletions within 22q11.2 are one of the most common microdeletions studied. We report a case of central 22q11.2 deletion with abnormal dentition, a feature not previously described in this condition. Although the diagnosis of central 22q11.2 deletion syndrome requires genetic testing, we aim to facilitate clinical recognition, expediting diagnosis.

## Graphical abstract

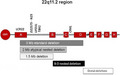

## 1 INTRODUCTION

**Figure 1:**
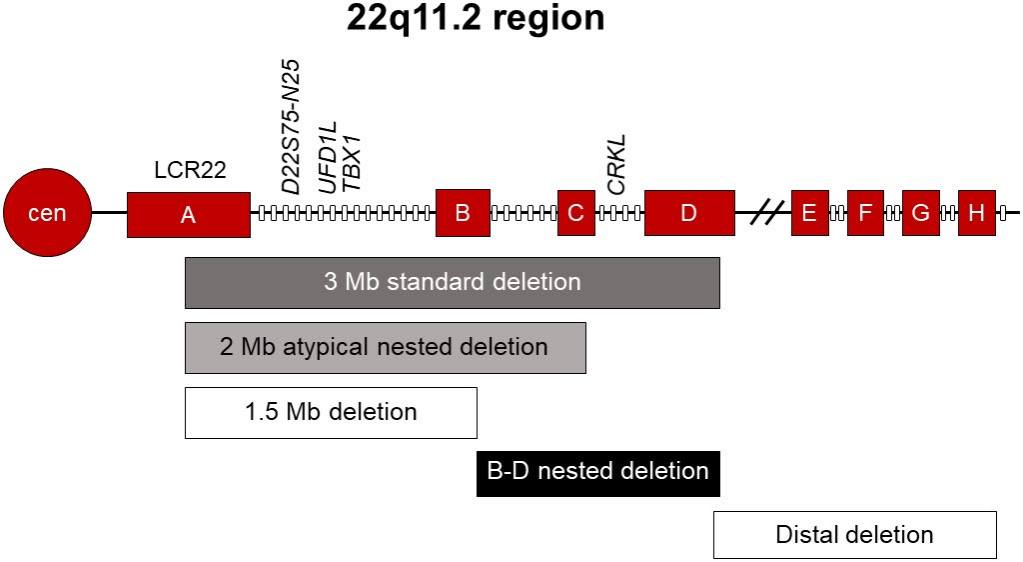
22q11.2 regions of deletion^2^

Chromosome 22q11.2 deletions are one of the most common and recognized microdeletions known with a prevalence of ~1 in 4000 live births.^1^ In 85% of affected individuals with a deletion at 22q11.2, an approximate 2.5‐Mb region is involved resulting in the loss of T‐box transcription factor (*TBX1*) and clinical features of DiGeorge syndrome/velo‐cardio‐facial syndrome.^2^ Microdeletions on the long arm of chromosome 22 are a result of nonallelic homologous recombination between areas of low copy repeat (LCR) sequences with the most common deletion occurring between the two largest regions LCR22‐A and LCR22‐D.^3^ Several various deletions have been described involving the regions A through H. Proximal deletions involve region A and can extend through H. Central, nested, or atypical deletions are commonly described as involving the B through E regions. Distal deletions involve regions E through H. Proximal deletions of the entire portion of LCR22‐A through LCR22‐D involving approximately 40 genes are found in 85% of affected individuals. The remaining 15% have smaller, atypical deletions. Atypical deletions involving LCR22‐C to D/E have been associated with a recognizable phenotype with characteristics including distinct facial features, congenital cardiac defects (including TOF), prematurity, pre‐ and/or postnatal growth restriction, microcephaly, and mild developmental delay.^4^ Penetrance is observed to be incomplete, and anticipation has not been detected.^2^ We present a case of central 22q11.2 deletion which does not involve the *TBX1* gene and exhibits a novel phenotype, abnormal dentition. In general, 22q11.2 deletions are associated with dysmorphic facies, intellectual disability and speech delay, hypotonia, CNS malformations, visual changes, behavioral issues, and, in one case, hydrops fetalis.^5^ Our patient has a 1.15 Mb microdeletion involving LCR22 B‐D [hg19] (20,312,560‐21,465,659) known as a B‐D nested deletion (Figure 1) and exhibits the novel phenotype of abnormal dentition.

## 2 CLINICAL REPORT/ CASE HISTORY

### 2.1 Background

Our patient is a 10‐year‐old male born prematurely to a 32‐year‐old gravida 10 para 2 mother via induced vaginal delivery at 35 weeks and 5 days due to oligohydramnios and decreased fetal movement. He was appropriate for gestational age with a birth weight of 2765 g (10th centile), and length was measured at 49 cm (30th centile).^6^ Complications during pregnancy included maternal asthma requiring steroid therapy, and the pregnancy was considered high‐risk due to recurrent miscarriages. Prenatal Quad screen was negative. There were no reported complications with his delivery. He was discharged home prior to 72 hours of age with his mother without any perinatal complications. Starting around six months of age, he was diagnosed with recurrent respiratory infections including RSV, pneumonia, and several middle ear infections resulting in placement of pneumatic equalizing tubes twice and removal of his adenoids. He had poor weight gain during the course of these illnesses and was diagnosed with failure to thrive. Gross motor, social, and fine motor skills were preserved. He walked at age 9‐10 months and demonstrated good tone and muscle bulk. Attempts to increase nutrition through solid foods, formula supplementation, and increased breastfeeding did not improve his weight velocity, leading to placement of a gastrostomy tube with Nissen fundoplication. With his recurrent infections and failure to thrive, workup for immunodeficiency and malabsorptive processes was initiated. Testing revealed negative celiac markers, normal electrolytes, and liver function, however, he was found to have an elevated sweat chloride level at 63 mmol/L. He was referred to Pulmonology to begin care for cystic fibrosis (CF). He initially responded well to treatment with high caloric nutrition and pulmonary treatments with improving weight gain; however, his suboptimal growth continued and further consultation was made with Endocrinology to consider other potential reasons for poor growth. Subsequent repeat sweat chloride tests were all found to be within normal limits and CF genetic testing was ultimately negative; therefore, alternate diagnoses were considered through molecular studies.

### 2.2 Clinical features

Aside from challenges related to growth, no distinctly abnormal clinical features were present until he had eruption of his primary teeth. The primary teeth were noted to be delayed and have abnormal morphology and described as brown in coloration and appeared “crumply.” When his secondary teeth erupted, they were noted to be peg shaped, pearly, and had a transparent appearance. Additional physical findings included a high‐arched palate, thin hair, and diminished sweating. Family history revealed several family members on the maternal side with dry skin described as “fish scales.” Our patient has two siblings who are reported to be unaffected without any similar clinical features.

Given concern for immunodeficiency with failure to thrive, abnormal dentition, thin hair, and decreased sweating, testing for ectodermal dysplasia and chromosomal microarray (CMA) was performed.

## 3 INVESTIGATION—MOLECULAR STUDIES

A chromosomal microarray was performed along with gene‐specific testing for CF and ectodermal dysplasia. The microarray showed a 1.15 Mb microdeletion involving chromosome 22 at LCR22 B‐D [hg 19] (20,312,560‐21,465,659). 22q11.2 deletion syndrome is a de novo mutation in 93% of reported cases with the remaining 7% inherited from a parent.^2^ The biological parents of our patient were tested and found not to harbor the deletion. Central deletions within the LCR22 B‐D region have been reported to be associated with several distinct phenotypes including: growth restriction (24%), developmental delay (24%), intellectual disability (25%), language delay (22%), immune deficiency (15%), palatal anomalies (7%), and dysmorphic features (46%). The most common dysmorphic features include abnormal ears, upslanting palpebral fissures, and a prominent forehead.^7^ At the time of this publication, the region deleted in our patient includes 16 OMIM identified genes which are listed in Table 1. There are no reported associations with these genes and abnormal dentition.^8^

**Table 1:** Protein coding OMIM Genes within 22q11.2 (22:20,312,560‐21,465,659)^8^

OMIM ID	OMIM Title	Mapped genes	GeneID
609459	DiGeorge Syndrome Critical Region Gene 6 Like	*DGCR6L*(22q11.21)	85359
194548	Zinc Finger Protein 74	*ZNF74*(22q11.21)	7625
613619	Scavenger Receptor Class F member 2	*SCARF2*(22q11.21)	91179
607372	Mediator Complex Subunit 15	*MED15*(22q11.21)	51586
600286	Phosphatidylinositol 4‐kinase Alpha	*PI4KA*(22q11.21)	5297
142360	Serpin Family D Member 1	*SERPIND1*(22q11.21)	3053
604202	Synaptosome‐Associated Protein 29	*SNAP29*(22q11.21)	9342
602007	CRK Like Proto‐oncogene, Adaptor Protein	*CRKL*(22q11.21)	1399
617298	Apoptosis‐Inducing Factor, Mitochondria Associated 3	*AIFM3*(22q11.21)	150209
600574	Leucine Zipper Like Transcription Regulator 1	*LZTR1*(22q11.1‐q11.2)	8216
609518	THAP Domain Containing 7	*THAP7*(22q11.21)	80764
608077	Purinergic Receptor P2X 6	*P2RX6*(22q11.21)	9127
603752	Solute Carrier Family 7 Member 4	*SLC7A4*(22q11.21)	6545
137181	Gamma‐glutamyltransferase 2	*GGT2*(22q11.21)	728441
612700	RIMS Binding Protein 3B	*RIMBP3B*(22q11.21)	440804
607712	HIC ZBTB Transcriptional Repressor 2	*HIC2*(22q11.21)	23119

Concern for ectodermal dysplasia was raised once our patient demonstrated the constellation of abnormal dentition, thin hair, and decreased sweating. Individuals with hypohidrotic ectodermal dysplasia classically demonstrate hypodontia (lack of dentition), hypotrichosis (lack of or thin hair), and hypohidrosis (impaired sweating) with physical and motor development being otherwise normal.^9^ For the evaluation of ectodermal dysplasia, a gene‐specific panel was obtained including: *BCS1L* (2q35), *CDH3* (16q22.1), *DSP* (6p24.3), *EDA* (Xq13.1), *EDAR* (2q13), *EDARADD* (1q42.3‐43), *ERCC2* (19q13.32), *EVC* (4p16.2), *EVC2* (4p16.2), *GJB2* (13q12.11), *GJB6* (13q12.11), *HOXC13* (12q13.13), *HR* (8p21.3), *IFT122* (3q21.3‐22.1), *JUP* (17q21.2), *KDF1* (1p36.11), *KREMEN1* (22q12.1), *KRT74* (12q13.13), *KRT85* (12q13.13), *LRP6* (12p13.2), *LTBP3* (11q13.1), *MPLKIP* (7p14.1), *MSX1* (4p16.2), *NFKBIA* (14q13.2), *PAX9* (14q13.3), *PORCN* (Xp11.23), *PRKD1* (14q12), *RMRP* (9p13.3), *TP63* (3q28), *WDR35* (2p24.1), and *WNT10A* (2q35).^10^ The panel was negative for any pathogenic variants.

For the evaluation of CF‐related gene mutations, testing investigating CFTR (7q31.2) mutations were obtained and were also found to be negative.^11^

## 4 DISCUSSION

Chromosomal deletions involving 22q11.2 regions have been well studied and described within the current literature. The patient discussed in this report presents with several findings common to 22q11.2 central deletion syndrome like failure to thrive, immunodeficiency, and palatal anomalies; however, he adds dysmorphic dentition to the reported phenotype. Using the Decipher Database, 33 individuals overlap with the deletion in our patient and demonstrated 28 distinct clinical features seen in Table 2 with full phenotype and region of deletion seen in Table 3. ^12^ As seen, abnormal, absent, or poor dentition has not been reported to date in patients with deletions inclusive to the region of chromosome 22 at LCR22 B‐D [hg 19] (20,312,560‐21,465,659).

**Table 2:** Aggregate data from Decipher Database of individuals with deletions overlapping 22q11 [hg 19] (20,312,560‐21,465,659)^12^

Reported Clinical Features for 22q11.2 Deletion Patients	
Behavioral	
Hyperactivity/Short Attention Span	4
Brain/CNS	
Hypotonia	4
Hypoplasia of corpus callosum	2
Hydrocephalus	1
Cardiac	
Cardiomyopathy	3
Supraventricular tachycardia	1
Connective Tissue	
Short Stature	3
Syndactyly/ Ligamentous laxity/ Redundant skin	3
Craniofacial	
Abnormal facies	11
Abnormal ear structure	7
Microcephaly/Craniosynostosis	6
Cleft Palate	1
Micrognathia	1
Developmental	
Intellectual Disability	8
Speech delay/Dysphasia	6
Global Developmental Delay	5
Growth retardation	2
Ocular	
Visual Impairment	3
Hypopigmentation of the fundus	1
Nystagmus	1
Strabismus	1
Other	
Seizure	2
Unilateral deafness	2
Scoliosis	1
Hydrops fetalis	1
Fetal cystic hygroma	1
Renal Agenesis	1
Rhabdomyolysis	1

**Table 3:** Phenotypic features and deletion coordinates of all 33 DECIPHER patients who overlap with 22q11.2 deletion (22:20,312,560‐21,465,659)^12^

Decipher Database Table				
Decipher ID	Variant‐del (22q11.2)	Sex	Inheritance	Phenotypic Features
273627	20716923‐21297749	46, XY	Unknown	Cleft palate, Facial asymmetry, Microtia, Preauricular skin tag, Stenosis of the external auditory canal, Unilateral deafness
289 202	20719137‐21441944	Unknown	Unknown	Global developmental delay, Myoclonic absences
260575	20721856‐21464119	46, XX	De novo constitutive	Abnormality of the nervous system, Abnormality of the palpebral fissures, Dysphagia, Global developmental delay, Inverted nipples, Redundant skin
262138	20721856‐21464119	46, XX	De novo constitutive	Abnormality of metabolism/homeostasis, Cardiomyopathy, Congenital hypothyroidism, Rhabdomyolysis, Specific learning disability
280907	20754422‐21368002	46, XX	Maternally inherited, constitutive in mother	Abnormal facial shape, Global developmental delay, Microcephaly
251336	20754422‐21382953	46, XX	Unknown	Intellectual disability, Seizures
257105	20754422‐21382953	46, XY	Inherited from normal parent	
264687	20754422‐21440514	46, XY	Unknown	
273516	20754422‐21440514	46, XX	Inherited from parent with unknown phenotype	Abnormality of the face, Hyperactivity
292621	20754422‐21440514	46, XY	Unknown	Delayed speech and language development
293486	20754422‐21440514	46, XY	Unknown	
300291	20754422‐21440514	46, XY	Unknown	Deep palmar crease
300741	20754422‐21440514	46, XX	Unknown	Generalized hypotonia, Hypopigmentation of the fundus, Intellectual disability, Ligamentous laxity, Nystagmus
304604	20754422‐21440514	46, XY	Unknown	Cortical visual impairment, Generalized hypotonia, Strabismus
332728	20754422‐21440514	46, XY	De novo constitutive	2‐3 toe syndactyly, Expressive language delay, Global developmental delay, Postnatal microcephaly, Short neck, Short stature
339286	20754422‐21440514	46, XY	Unknown	Behavioral abnormality
340049	20754422‐21440514	46, XX	Unknown	Hypermetropia, Specific learning disability
271760	20754451‐21440484	46, XY	Unknown	
262738	20958984‐21382953	46, XX	De novo constitutive	Almond‐shaped palpebral fissure, Congenital microcephaly, Craniosynostosis, Highly arched eyebrow, Hypoplasia of the corpus callosum, Intellectual disability, Micrognathia, Muscular hypotonia, Renal agenesis, Ridged cranial sutures
249399	21032298‐21449852	46, XX	Unknown	Intellectual disability, Short attention span
251146	21060358‐21461607	46, XY	Unknown	Downslanted palpebral fissures, Facial asymmetry, Proportionate short stature, Round face
279514	21067691‐21465659	46, XY	Unknown	Dilated cardiomyopathy, Supraventricular tachycardia
249400	21075319‐21449852	46, XX	Unknown	Hyperactivity
253463	21075575‐21368002	46, XY	Unknown	
261441	21075575‐21440514	46, XY	Inherited from parent with similar phenotype to child	
287105	21075575‐21440514	46, XX	De novo constitutive	Intrauterine growth retardation, Postnatal growth retardation
339858	21075575‐21464119	46, XX	Maternally inherited, constitutive in mother	Fetal cystic hygroma, Hydrops fetalis
331632	21076930‐21441944	46, XY	De novo constitutive	Intellectual disability, Short stature
263251	21078946‐21460598	46, XY	Inherited from normal parent	Upslanted palpebral fissure
255 749	21095275‐21464119	46, XY	Inherited from normal parent	Abnormality of the middle ear ossicles, Abnormality of the pinna, Delayed speech and language development, Intellectual disability, Myopia, Prominent ear helix, Unilateral deafness
284733	21134126‐21440514	46, XY	Paternally inherited, constitutive in father	Abnormal heart morphology, Hydrocephalus, Intellectual disability, moderate
357693	20733427‐21464119	46, XY	Paternally inherited, constitutive in father	Asymmetry of the ears, Delayed fine motor development, Delayed gross motor development, Scoliosis, Skull asymmetry
359383	20716876‐2143141	46, XY	Unknown	Specific learning disability

The abnormal dentition reported in our patient along with thin hair and decreased sweating can be seen in patients with ectodermal dysplasia and may be unrelated to the patient's microdeletion. However, current available panel testing was negative for mutations with known association with ectodermal dysplasia. In addition, the genes currently implicated in ectodermal dysplasia (chrs 1, 2, 4, 11, 14, and X) are not located within the 22q11.2 deletion found in our patient.

## 5 CONCLUSION

The patient presenting within this case report harbors a microdeletion within a common region on the 22nd chromosome but offers a novel phenotype of dysmorphic dentition. Several other clinical findings suggested a diagnosis of ectodermal dysplasia; however, gene‐specific testing was negative. Therefore, this novel phenotype presented may be either a novel physical characteristic of central 22q11.2 deletion syndrome, an unrecognized ectodermal dysplasia variant, or, possibly, unrelated to either. By adding this clinically relevant feature of abnormal dentition to the literature, we aim to expedite clinical recognition and improve prognostication for individuals affected by 22q11.2 deletion syndrome.

## CONFLICT OF INTEREST

None declared.

## AUTHOR CONTRIBUTIONS

Patrick Dideum MD: involved in data collection and the manuscript author. Luis Rohena MD: involved in manuscript editing and the managing geneticist. Janet Berg RN: involved in data collection and interpretation and the managing genetic and metabolic nurse. Candace Percival MD: involved manuscript editing and the managing endocrinologist.
